# Long-Term Progression and Rapid Decline in Hearing Loss in Patients with a Point Mutation at Nucleotide 3243 of the Mitochondrial DNA

**DOI:** 10.3390/life12040543

**Published:** 2022-04-06

**Authors:** Aki Sakata, Akinori Kashio, Hajime Koyama, Tsukasa Uranaka, Shinichi Iwasaki, Chisato Fujimoto, Makoto Kinoshita, Tatsuya Yamasoba

**Affiliations:** 1 Department of Otolaryngology and Head and Neck Surgery, Graduate School of Medicine, The University of Tokyo, Tokyo 113-8654, Japan; harvest-tky@umin.ac.jp (A.S.); kashioa-tky@umin.ac.jp (A.K.); hakoyama-tky@umin.ac.jp (H.K.); uranakat-oto@h.u-tokyo.ac.jp (T.U.); iwashin@med.nagoya-cu.ac.jp (S.I.); cfujimoto-tky@umin.ac.jp (C.F.); kinoshitam-zao@umin.ac.jp (M.K.); 2 Department of Otolaryngology and Head and Neck Surgery, Graduate School of Medicine, Nagoya City University, Aichi 467-8601, Japan

**Keywords:** hearing loss, dizziness, disequilibrium, diabetes, mitochondrial gene mutations

## Abstract

Patients with m.3243A>G mutation of mitochondrial DNA develop bilaterally symmetric sensorineural hearing loss. However, it is unclear how fast their hearing loss progresses over time, and whether they experience rapid progression of hearing loss. In the present study, we conducted a long-term hearing evaluation in patients with MELAS or MIDD who harbored the m.3243A>G mutation of mitochondrial DNA. A retrospective chart review was performed on 15 patients with this mutation who underwent pure-tone audiometry at least once a year for more than two years. The mean follow-up period was 12.8 years. The mean progression rate of hearing loss was 5.5 dB per year. Hearing loss progressed rapidly to be profoundly deaf in seven patients during the observation period. Heteroplasmy and age-corrected heteroplasmy levels correlated with the age of onset of hearing loss. These results indicate that patients with m.3243A>G mutation have a gradual progression of hearing loss in the early stages and rapid decline in hearing to be profoundly deaf in approximately half of the patients. Although it is possible to predict the age of onset of hearing loss from heteroplasmy and age-corrected heteroplasmy levels, it is difficult to predict whether and when the rapid hearing loss will occur.

## 1. Introduction

Sensorineural hearing loss is frequently associated with mitochondrial disease; it is observed in approximately half of the patients with three main syndromes—chronic progressive external ophthalmoplegia (CPEO); myoclonus epilepsy associated with ragged-red fibers (MERRF); mitochondrial encephalomyopathy, lactic acidosis, and stroke-like episodes (MELAS) [1]. Hearing loss mainly involves the cochlea in mitochondrial diseases, but it sometimes accompanies central auditory abnormalities [1,2,3,4,5].

Mitochondrial diseases are categorized into two groups: those characterized by ragged-red fibers (RRF), including MELAS, MERRF, and CPEO, and those caused by mutations in protein-coding genes, such as pure encephalopathy without RRF. RRF is a characteristic microscopic appearance of the muscle stained with Gomori trichrome, which is due to the accumulation of abnormal mitochondria below the plasma membrane of the muscle fiber. Hearing loss is highly prevalent in the first group but rarely seen in the second group. Oxidative phosphorylation is impaired in both types of encephalopathy; however, mitochondrial protein synthesis is impaired only in the first group, suggesting that hearing loss is caused by impairment of protein synthesis similar to RRF.

An A-to-g transition mutation at nucleotide pair (np) 3243 in mitochondrial DNA (mtDNA) has been documented in most patients with MELAS and a few patients with CPEO. This mutation has been identified in pedigrees with maternal transmission of diabetes and deafness among families of different racial backgrounds [6,7,8,9,10]. It has been reported that patients with higher heteroplasmy levels of this point mutation exhibit MELAS [11,12], while those with low heteroplasmy levels mainly develop hearing loss and diabetes; those with a low heteroplasmy level have maternally inherited diabetes and deafness (MIDD) [9].

In patients with MELAS and MIDD, hearing is generally normal at birth, and hearing loss occurs eventually, appearing as early as in the teens or twenties and as late as the fifties. Hearing loss, in general, is bilateral and symmetrical; a pure-tone audiogram shows a flat type or falling type of sensorineural loss bilaterally in most cases [6,9]. Hearing loss mainly involves the cochlea [6,13,14]; however, when the disease progresses, retro-labyrinthine and central auditory pathways are also affected [15,16]. In addition to hearing loss, the vestibular system is also involved, and patients eventually suffer from impairment of balance and gait [6,17,18,19].

We previously reported that, when present for several years, patients with np3243 point mutation showed a slightly progressive decline in their hearing; the progression rate of hearing loss ranged from 1.5 to 7.9 dB per year [7]. However, it is unknown how rapidly their hearing loss progresses during the long-term period, and whether there is a rapid progression of hearing loss. Therefore, in the present study, we evaluated their hearing in the long-term period in patients with MELAS or MIDD who harbored m.3243A>G mutation of mtDNA. We also investigated the relationship between heteroplasmy and age-corrected heteroplasmy levels and the age of onset of hearing loss and the progression rate of hearing loss, as well as the relationship between the age of onset of hearing loss, diabetes, and balance disorder.

## 2. Materials and Methods

### 2.1. Patients

In total, 27 patients with hearing loss and an A-to-g transition at np 3243 in the mtDNA visited the Department of Otolaryngology and Head and Neck Surgery, at the University of Tokyo Hospital, between 1989 and 2021. Among them, 15 patients who underwent audiological examinations for more than two years or until they became completely deaf were enrolled. The patients consisted of four males and eleven females, and their ages ranged from 22 to 66 years (mean: 40 years) at their first visit to our clinic. All patients had hearing loss, with a pure-tone average (PTA) value being greater than 25 dB HL (hearing level) on the initial audiological examination. They were interviewed regarding the onset of hearing loss, balance–gait disorder, diabetes mellitus, and the presence of other signs and symptoms. The presence of diabetes mellitus was confirmed by doctors in the Department of Nutrition and Metabolism of our hospital or their affiliated hospital, and the blood glucose and HbA1c levels were periodically measured in all patients. All procedures were in accordance with the Helsinki declaration and were approved by the University of Tokyo Human Ethics Committee (No. 2487). All patients gave informed consent for the use of their clinical data.

### 2.2. Audiological and Neuro-Otological Evaluation

The patients were evaluated using pure-tone audiometry, in general, every three months, to assess the longitudinal changes in their hearing thresholds. The pure-tone average (PTA) values were calculated as the mean air conduction threshold at 0.5, 1, 2, and 3 kHz. The pure-tone threshold at 3 kHz was obtained from the mean of 2 and 4 kHz, as it is not routinely measured in Japan. For calculating the PTA values, the hearing thresholds at the frequencies showing off-scale were calculated as 5 dB over the maximum sound level generated by an audiometer. The patients were also evaluated using speech recognition test, tympanometry, acoustic reflex threshold test, auditory brainstem response, and distortion-product otoacoustic emissions (DPOAEs), when necessary.

The patients also underwent a battery of neuro-otological evaluations consisting of a physical examination, neurological examination, and neuro-otological examinations, including caloric and cervical vestibular evoked myogenic potential (cVEMP) testing.

Caloric testing was performed with 2 mL ice water irrigation of the external auditory canal for 20 s. This caloric stimulation method is easier to perform than bithermal irrigation and has high sensitivity and specificity for detecting canal paresis [20]. Electronystagmography was used to record the induced nystagmus while the subject lay supine with their head raised at an angle of 30 degrees. The percentage of canal paresis was calculated as 100 × |(MSEVr − MSEVl)/(MSEVr + MSEVl)|, where MSEVr is the maximum slow-phase eye velocity of the right side, and MSEVl is that of the left side. A value of canal paresis >20% was regarded as abnormally reduced on the affected side [21]. When MSEVr and MSEVl were <10 degrees/s, it was regarded as a reduced response on both sides [22].

In the role of a stimulus for cVEMP, short tone bursts of 500 Hz (95 dB normal hearing level; 135 dB SPL (peak value); rise/fall time, 1 ms; plateau time, 2 ms) were presented through headphones (type DR-531; Elega Acoustic Ltd., Tokyo, Japan). Surface electromyographic activity was recorded in supine patients from symmetrical sites over the upper half of each sternocleidomastoid muscle (SCM), with a reference electrode on the lateral end of the upper sternum. During recording, the patients were instructed to raise their heads slightly to continuously contract the SCM. Background EMG was monitored during recording to confirm that subjects maintained SCM activity at a sufficient level (150 μV). We analyzed the first biphasic wave (p13-n23) from the ipsilateral SCM to the stimulated side. For the evaluation of amplitude. The percentage of cVEMP asymmetry ratio (cVEMP AR) was calculated as 100|(Ar − Al)/ (Ar + Al)|, where Ar is the amplitude of p13-n23 on the right side, and Al is the amplitude of the p13-n23 on the left side. On the basis of results from normal subjects, the upper limit of cVEMP AR was set to 34.0 [23]. We regarded it as an “absent” response when no reproducible p13-n23 was present in two runs. We regarded it as a “reduced” response when a reproducible p13-n23 was present, and the AR was greater than the predefined upper limit for normal subjects. We regarded it as bilaterally abnormal responses when the response was absent on both sides.

### 2.3. DNA Studies

The invader assay contract by BML (Tokyo, Japan) was applied for the screening of mitochondrial tRNA (Leu). Briefly, mtDNA isolated from the peripheral leukocytes of patients and 1.2 μL of primary probe/invader oligonucleotides mixture (containing 0.5 umol/L wild-type primary probes, 0.5 umol/L mutant primary probes, 0.05 umol/L invader oligonucleotide, and 10 mmol/L 3-(N-morpholino) propanesulfonic acid) were poured into each well of the plates. Fluorescent resonance energy transfer (FRET)/Cleavase mixture (Hologic, Marlborough, MA, USA) was added to the probe/invader oligonucleotide-containing plates. Subsequently, 3 μL of 5–100 mol/L synthetic target oligonucleotides (positive control), 10 ug/mL yeast tRNA (no target control), and denatured genomic DNA samples (>15 ng/μL) were added. Next, 6 μL of mineral oil (Sigma, St. Louis, MO, USA) was overlayed into all reaction wells and incubated at 63 °C for 4 h. After incubation, the fluorescence was measured using a Cyto Fluor 4000 fluorescent microplate reader. The heteroplasmy rate for mitochondrial mutations was quantified by the detection of fluorescently labeled and digested PCR products through a fluorescence imaging system [24,25]. The age-corrected heteroplasmy level in leucocytes was calculated using the following formula: (leucocyte heteroplasmy)/0.977^(age+12)^. This correction was previously published by Grady et al. [26]. The current paper refers to this value as the age-corrected heteroplasmy level in leucocytes.

### 2.4. Statistical Analysis

The correlation coefficients were calculated to assess the relationship between the heteroplasmy and age-corrected heteroplasmy levels and the progression rate of hearing loss and between the heteroplasmy and age-corrected heteroplasmy levels and the onset of hearing loss, balance or gait disorder, and diabetes mellitus, using JMP 16 (SAS Institute, Cary, NC, USA). The age of onset of hearing loss, diabetes, and balance disorder was compared against each other using the Tukey–Kramer test. We performed the Shapiro–Wilk W test to value the normality of the sample. The relationship between the two continuous variables was analyzed by single regression analysis. R2 represented the coefficient of determination, and the significance of the slope indicated the *p*-value. *p* < 0.05 was considered statistically significant.

## 3. Results

The patients’ demographic characteristics of the patients are shown in Table 1. The heteroplasmy levels ranged from 3% to 37%, with the mean being 23.9%. In terms of gender, 4 patients were male, and 11 were female. Most patients had bilaterally symmetric hearing loss with a horizontal or sloping type of audiogram. The mean age of onset of hearing loss was 28.6 years, with acquired hearing loss being perceived as early as 10 years of age in the earliest cases and as late as 56 years of age in the latest cases. At their first visit, 13 patients had diabetes mellitus, 3 had cerebellar atrophy or stroke on head MRI, and 1 had a cardiac disease; two patients eventually developed diabetes during the follow-up period. Other than hearing loss and diabetes mellitus, 11 patients did not show any symptoms or signs suggestive of MELAS.

**Figure 1:**
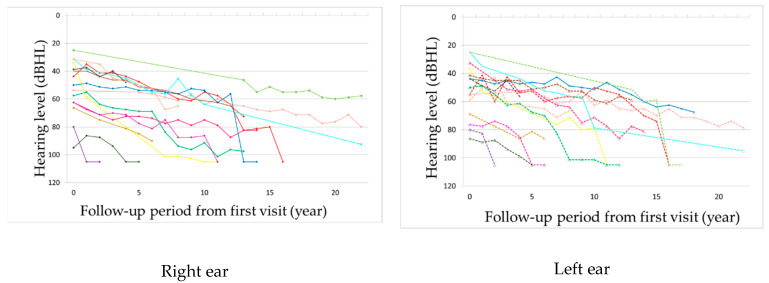
Chronological progression of hearing level from the first visit. The different color indicates the different patient, and the same color indicates the same patient.

**Figure 2:**
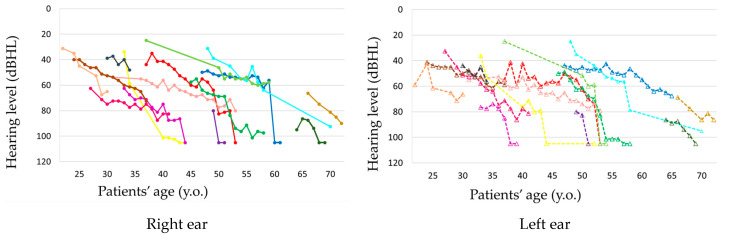
Relationship between patients’ age and progression of their hearing loss. The different color indicates the different patient, and the same color indicates the same patient.

**Figure 3:**
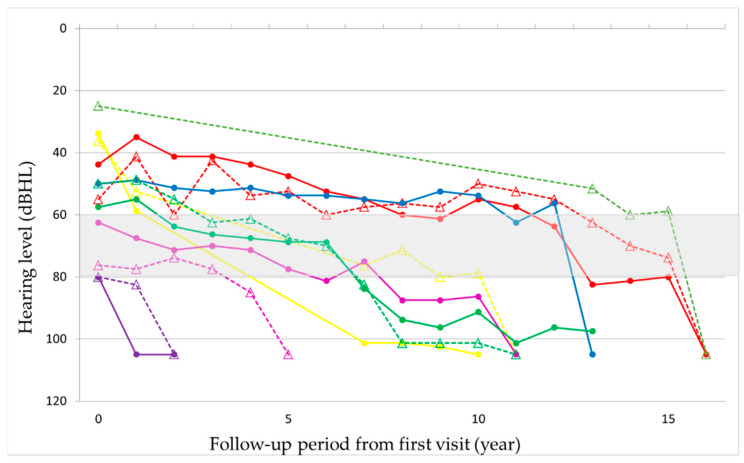
Chronological change in hearing level from the first visit in patients who showed a rapid decline in hearing. The different color indicates the different patient, and the same color indicates the same patient. The solid lines with the shaped mark: right ear. The dashed line with the triangle mark: left ear. Two patients (light green and blue) only have one ear shown because the rapid decline was only seen in one ear.

The observation period ranged from 2 to 22 years, with a mean of 12.8 years. Hearing loss progressed in all patients during the observation period (Figure 1 and Figure 2, Supplementary Figures S1 and S2). In seven patients, hearing deteriorated rapidly to complete deafness from 40 to 63 years of age (mean: 50 years). This episode was not associated with the worsening of pre-existing signs such as diabetes or other symptoms or signs. Before the rapid progression of hearing loss, their hearing level ranged from 56 to 80 dB HL. The rapid progression occurred on both ears almost simultaneously in three patients and at different times in two patients, with the periods between the ears being 1 and 7 years, respectively, and only on one ear in two patients (Figure 3 and Supplementary Figure S3). Although all these patients were treated with oral or systemic steroids, their hearing did not show any improvement. The heteroplasmy level ranged from 9% to 30% (mean: 20%) in seven patients, who showed rapid progression of hearing loss, and from 3% to 37% (mean: 23%) in the remaining eight patients, with no significant difference between them. The age-corrected heteroplasmy level ranged from 18.4% to 95.7% (mean: 68.6%) in seven patients, who showed rapid progression of hearing loss, and from 20.2% to 81.6% (mean: 64.4%) in the remaining eight patients, with no significant difference between them.

The mean rate of the progression of hearing loss in all 15 patients was 5.5 dB per year. In eight patients, who did not show rapid deterioration of hearing, the progression rate of hearing loss was 3.0 dB per year, while it was 1.9 dB per year before the rapid deterioration in seven patients. When the progression rate of hearing loss prior to the rapid deterioration in the latter was added to the calculation, the mean progression rate of hearing loss was 2.5 dB per year in all patients. When the progression rate of hearing loss prior to the rapid deterioration in the latter was added to the calculation, the mean progression rate of hearing loss was 2.5 dB per year in all 15 patients and did not differ significantly between the patients with and without rapid deterioration in hearing.

Caloric and cVEMP tests were performed in 13 out of 15 patients. In the caloric test, 4 (30%) out of 13 patients showed unilaterally decreased response, and 6 (46%) showed bilaterally decreased response. In cVEMP, 6 (46%) out of 13 patients showed unilateral abnormalities, and 7 (54%) showed bilateral abnormalities.

**Figure 4:**
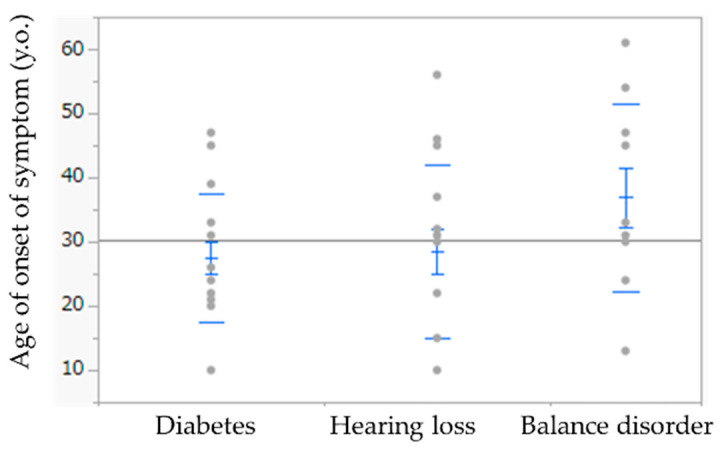
Age of the onset of diabetes, hearing loss, and balance disorder. Blue upper long bar, upper whisker; blue upper short bar, upper quartile; blue center short bar, median; blue lower short bar, lower quartile; blue lower long bar, lower whisker.

The mean age of onset of hearing loss, diabetes, and balance disorder was 28.6, 27.5, and 36.9 years, respectively (Figure 4). Based on the Shapiro–Wilk test, the *p*-values for the age of onset of hearing loss, diabetes, and balance disorder were 0.269, 0.400, and 0.189. The onset of balance disorder was delayed, compared with hearing loss and diabetes, but the difference was not statistically significant. However, balance disorder did not manifest in five patients until the end of the observation period; if we assume that the balance disorder appeared in these five patients one year later than the end of the observation period, the difference would be statistically significant (*p* = 0.0092 vs. diabetes; *p* = 0.0161 vs. hearing loss).

**Figure 5:**
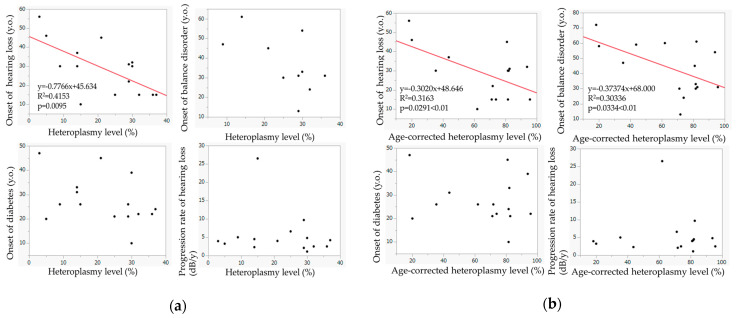
(**a**) Relationship between heteroplasmy level and the onset of hearing loss, balance–gait disorders, and diabetes mellitus and the progression rate of hearing loss; (**b**) relationship between age-corrected heteroplasmy level and the onset of hearing loss, balance disorders, and diabetes mellitus and the progression rate of hearing loss. The red lines: the regression lines.

Figure 5a shows the relationship between the heteroplasmy level and the ages of onset of hearing loss, diabetes mellitus, and balance–gait disorder and between the heteroplasmy levels and the progression rate of hearing loss. The heteroplasmy levels showed a significant relationship with the onset of hearing loss (*p* = 0.0095); hearing loss appeared at a younger age in patients with higher heteroplasmy levels. Such a trend was also observed in the relationship between the heteroplasmy levels and the onset of balance disorder. The heteroplasmy levels were not associated with the onset of diabetes or the progression rate of hearing loss.

Figure 5b shows the relationship between the age-corrected heteroplasmy level and the ages of onset of hearing loss, diabetes mellitus, and balance–gait disorder, and between the heteroplasmy level and the progression rate of hearing loss. Age-corrected heteroplasmy levels showed significant correlations with the onset of hearing loss (*p* = 0.0291) and the onset of balance disorder (*p* = 0.0334); balance disorder appeared at a younger age in patients with higher age-corrected heteroplasmy. Age-corrected heteroplasmy level was not associated with the onset of diabetes or the progression rate of hearing loss.

## 4. Discussion

The current study evaluated the hearing in 15 patients with m.3243A>G mutation of mtDNA in the long-term period from 2 to 26 years (mean: 12.8 years) after their first hearing test. The mean age of onset of hearing loss was 28.6 years; hearing loss occurred between 10 and 56 years old. The age of onset of hearing loss was correlated with the heteroplasmy and age-corrected heteroplasmy levels. Initially, from the start of their follow-up, their hearing loss progressed gradually, but the hearing loss progressed rapidly to deafness in seven patients during the observation period. The hearing level ranged from 56 to 80 dB HL before the rapid deterioration of hearing. The progression rate of hearing loss before the rapid deterioration in these patients did not significantly differ from that in the remaining eight patients who did not show rapid hearing deterioration. All these results indicate that it is difficult to predict the rapid hearing decline in patients with m.3243A>G mutation of mtDNA. Oral or systemic steroid treatment was not effective in improving hearing loss.

Unlike m.1555A>G mutation, hearing loss caused by m.3243A>G mutation of mtDNA is progressive. When we first reported the audiological findings of five patients with this mutation in 1996, the progression rate of hearing loss ranged from 1.5 to 7.9 dB per year [8]. Since other studies have not reported the progression rate of hearing loss, it has not been unclear how rapidly the hearing declines in patients with the m.3243A>G mutation. In the current study, the progression rate of hearing loss ranged from 1.1 to 26.5 dB per year, with a mean of 5.5 dB per year. The progression rate of hearing loss was 3.0 dB per year in eight patients without rapid deterioration of hearing and 1.9 dB per year prior to the rapid deterioration in the remaining seven patients. When only the progression rate of hearing loss prior to the rapid deterioration in the latter was added in the calculation, the mean progression rate of hearing loss was 2.5 dB per year in all patients. This progression rate of hearing loss is more rapid than that seen in elderly subjects with age-related hearing loss. Therefore, patients with m.3243A>G mutation should be informed that their hearing loss will progress by an average of 25 dB after 10 years and advised to start wearing hearing aids at an early stage and structure their future living environment.

In the current study, the rapid progression of hearing loss occurred in 7 out of 15 patients, in both ears almost simultaneously in 3 patients, at different times in 2 patients, and only on one ear in 2 patients. This finding is quite interesting since such a rapid decline in hearing has not been reported except in one study. Oshima et al. (1996) reported the case of a 35-year-old woman with a complaint of right hearing loss and tinnitus in whom the pure-tone audiogram demonstrated 40 dB flat-type sensorineural hearing loss on the left and 85 dB saucer-type sensorineural hearing loss on the right ear [9]. Although the hearing at middle frequencies on the right ear improved after oral administration of steroids, it fluctuated and did not respond to oral administration of steroids or glycerol thereafter. Since hearing did not improve by oral or systemic administration of steroids in any of our patients, the pathophysiology of the fluctuating hearing in the case reported by Oshima et al. [9] is likely different from that of rapid hearing progression in our patients.

In our case series, the rapid decline in hearing occurred after the hearing loss exceeded 55 dB HL. Prior to the rapid progression of hearing loss, their hearing level ranged from 56 to 80 dB HL. It is unclear why such a rapid decrease in hearing occurred. Since the rapid decline in patients’ hearing was not associated with worsening of the pre-existing signs and symptoms or other signs and symptoms, it is unlikely that it was caused by a rapid decline in systemic mitochondrial function, at least in the four patients who had rapid hearing loss in both ears at different times or only in one ear. It is possible that the vascular supply to the cochlea was compromised due to diabetes mellitus or the stroke-like episode seen in MELAS. It is also possible that the endolymphatic potential was rapidly reduced due to the acute impairment of energy production in the stria vascularis.

Human temporal bone histopathological studies in patients with MIDD and MELAS showed that the stria vascularis most severely degenerated [27,28,29]; in a patient with MIDD, there was marked degeneration of the stria vascularis and outer hair cells throughout the cochlea, as well as a reduction in the number of spiral ganglion cells at the base [27]. Severe degeneration of the stria vascularis and degenerative change in the spiral ganglion cells were observed in two patients with MELAS, in whom quantitative DNA studies showed that the proportion of mutant to wild-type mtDNA was similar in both histologically affected and unaffected tissues within the inner ear. Long-term administration of germanium dioxide causes renal failure, emaciation, and muscle weakness in humans [30,31] and body weight loss, myopathy, and nephropathy in rats. The skeletal muscles of rats treated with germanium dioxide showed numerous ragged-red fibers, cytochrome c oxidase-deficient fibers, and the accumulation of electron-dense material in the mitochondria [30,32,33], which resembles the pathological findings observed in patients with mitochondrial encephalomyopathy. We previously reported that guinea pigs fed chows containing 0.5% germanium dioxide for 2 months developed hearing loss, mainly due to the degeneration of the stria vascularis and cochlear supporting cells, and exhibited decreased cytochrome c oxidase activity in the skeletal muscles and kidney [34]. No apparent pathological changes were observed in the utricle, semicircular canal, or the cochlear or vestibular nerve fibers, indicating that germanium dioxide-induced mitochondrial dysfunction mainly affects the stria vascularis and supporting cells in the cochlea, as in the skeletal muscles and kidney, causing hearing impairment in the guinea pigs. This animal study also supported the importance of mitochondrial function in the stria vascularis for the maintenance of hearing function.

In the present study, patients noted balance–gait disorder later, compared with hearing loss, and five patients were not aware of balance or gait disorder. Balance–gait disorder tended to appear later, compared with hearing loss and diabetes mellitus. The use of cVEMP is essential in the diagnosis of saccular dysfunction in patients with moderate-to-profound sensorineural hearing loss [35]. The low metabolic rate of the vestibular apparatus, compared with that of the stria vascularis, may make the vestibule more resistant to mtDNA mutations, leading to a later onset of vestibular dysfunction [21]. However, in 13 patients who underwent caloric and cVEMP tests, 4 (31%) and 6 patients (46%) showed decreased response unilaterally and bilaterally, respectively, in the caloric test, and 6 (46%) and 7 patients (54%) showed abnormalities unilaterally and bilaterally, respectively, in the cVEMP test. The caloric test evaluates the function of the lateral semicircular canal and the superior vestibular nerve, and the cVEMP evaluates the function of the saccule and the inferior vestibular nerve. Therefore, the vestibular systems, semicircular canals, and otolith organs are also frequently involved in patients with m.3243A>G mutation. Balance and gait disturbances may not become apparent until the vestibular function is severely impaired. It is interesting to note that seven patients with m.3243A>G mutation who had abnormal findings in the caloric and cVEMP tests showed normal responses in the galvanic VEMP test [18], which indicates that the peripheral vestibular end-organs are primarily affected similarly to the auditory system.

Human temporal bone histopathological studies on MIDD and MELAS demonstrated conflicting findings in terms of the degeneration of the vestibular systems. In a patient with MIDD, the vestibular end-organs, including the utricle and semicircular canals, were well preserved [6], whereas two patients with MELAS showed degenerative changes in the vestibular end-organs and Scarpa’s ganglions [29]. In one of these MELAS patients, there was a pathological collapse of the membranous wall of the saccule and significant hair cell loss in the saccular macula, utricular maculae, and the cristae of all three semicircular canals [29]. In another patient with MELAS, the hair cells in both cristae and maculae were reduced, and the numbers of Scarpa ganglion cells were reduced to approximately 70% of the mean counts of age-matched control samples [29].

It has been reported that the disease severity and the expression pattern of the impairment across organs and tissues may vary in mitochondrial diseases, depending on the heteroplasmy levels. In general, patients with a higher heteroplasmy level exhibit more severe phenotypes, although the correlation may be weak [26,36,37,38]. Since the heteroplasmy level in the inner ear cannot be assessed clinically, we adopted the heteroplasmy level in peripheral leucocytes and found that the heteroplasmy level correlated with the onset of hearing loss; hearing loss developed at a younger age in patients with higher heteroplasmy. Previous studies also demonstrated that hearing loss developed earlier in patients with higher heteroplasmy levels [7,11]. The onset of balance–gait disorder correlated weakly with heteroplasmy level but significantly with age-corrected heteroplasmy level. The heteroplasmy level and age-corrected heteroplasmy level did not correlate with the onset of diabetes mellitus. Iwasaki et al. (2011) reported no correlation between heteroplasmy and onset of balance or gait disorder in 13 unrelated patients with m.3243A>G mutation [18]. Other studies showed a negative correlation between the onset of diabetes and heteroplasmy [11,39]. These discrepancies may be due to the small sample sizes, different phenotypes, and different stages of disease among reports. For example, in the current study, only patients with complaints of hearing loss were enrolled; therefore, there were two patients who did not have diabetes mellitus at the time of initial examination. Such bias in patient selection may have resulted in different correlations between the heteroplasmy levels and the onset of diabetes mellitus or balance–gait disorder.

It is worthy to note that the heteroplasmy and age-corrected heteroplasmy levels did not correlate with the progression rate of hearing loss or the presence of a rapid decline in hearing in our cases. It is unclear whether such a trend is also observed in other organs or tissues. De Laat et al. [36] scored disease severity using the Newcastle Mitochondrial Disease Adult Scale (NMDAS), including SF-36 quality of life (QoL) scores, and measured heteroplasmy levels in urinary epithelial cells, leucocytes, and saliva in 151 carriers of m.3243A>G mutation of mtDNA; their results indicate a yearly increase in NMDAS score of 0.47 point in the total group and that heteroplasmy levels in both leucocytes and urinary epithelial cells were only weakly correlated with disease severity. They also observed that physical QoL declined with age and that the most important determinants of QoL decline were hearing loss, speech problems, exercise intolerance, gait instability, psychiatric problems, and gastrointestinal involvement.

No medication is known to prevent or slow the progression of hearing loss associated with m.3243A>G mutation of mtDNA. Since the hearing loss involves mainly the cochlea, hearing aids are effective until the hearing loss becomes severe to profound. When hearing aids become ineffective for oral communication, cochlear implantation, which directly stimulates the auditory nerve, is recommended [40]. In the present study, six patients received a cochlear implant at our department, and all patients except one who developed cerebral stroke 5 months after the cochlear implant surgery achieved good speech perception. As mentioned above, temporal bone histopathological studies demonstrated that the spiral ganglion cells were relatively well preserved in a patient with MIDD and degenerated in patients with MELAS, whereas the stria vascularis was severely degenerated [26,28]. Since hearing thresholds mainly reflects the functions of the cochlear cells, such as the hair cells and stria vascularis, it is presumed that the retro-cochlear auditory pathways, including the spiral ganglion cells, are still relatively well preserved when patients become profoundly deaf, which explains the high efficacy of cochlear implants. However, during long-term follow-up in the patients who have cochlear implantation with mitochondrial gene mutation, some patients who initially showed good speech perception exhibited deterioration of speech perception [41]. Therefore, patients receiving cochlear implantation should be carefully monitored over the long term.

## 5. Conclusions

This study evaluating the hearing in 15 patients with m.3243A>G mutation in mtDNA in the long-term period demonstrated that hearing loss occurred from 10 to 56 years of age, that the age of onset of hearing loss was correlated with heteroplasmy and age-corrected heteroplasmy levels, that the hearing loss progressed gradually initially, and that the rapid decline in hearing loss to profound deafness occurred in approximately half of the patients during the observation period. The hearing level prior to the rapid decline ranged from 56 to 80 dB HL, and the progression rate of hearing loss prior to the rapid decline was not significantly different from that in the remaining patients, who did not experience a rapid decline in hearing. Heteroplasmy and age-corrected heteroplasmy levels did not correlate with the presence of rapid progression of hearing loss or with the progression rate of hearing loss. These results indicate that it is difficult to predict the rapid decline in hearing in patients with m.3243A>G mutation of mtDNA. Neither oral nor systemic steroid treatment was effective in improving hearing loss.

## Figures and Tables

**Table 1:** Demographic characteristics of the patients.

Heteroplasmy Level	Age-Corrected Hetero-Plasmy Level	Gender	Onset of Hearing Loss (y.o.)	Right HL at First Visit (dBHL)	Left HL at First Visit (dBHL)	Progression Rate of Hearing Loss (dB/yr)	Rapid Decline of Hearing	CI	Onset of Balance Disorder (y.o.)	Caloric Test	cVEMP	Onset of Diabetes (y.o.)	MIDD/MELAS
37	81.6	F	15	31.3	58.8	4.2				NE	NE	24	MELAS
36	95.7	M	15	38.8	43.8	2.5		y	31	UNI-H	UNI-N.R.	22	MIDD
32	74.0	F	15	40	41.3	2.5			24	NOR	UNI-N.R.	22	MIDD
30	93.8	F	32	43.8	55	4.8	y	y	54	NOR	UNI-N.R.	39	MIDD
30	81.6	F	30	53.8	55	1.1			33	UNI-H	BI-N.R.	10	MIDD
29	82.6	F	31	33.8	36.3	9.7	y	y	31	UNI-H	UNI-N.R.	21	MIDD
29	71.9	F	22	62.5	32.5	2.1			13	BI-H	BI-N.R.	26	MIDD
25	71.2	F	15	62.5	76.3	6.6	y		30	BI-H	BI-N.R.	21	MELAS
21	81.0	F	45	57.5	50	4	y	y	45	BI-H	BI-N.R.	45	MIDD
15	62.0	F	10	80	80	26.5	y	y		BI-H	BI-N.R.	26	MIDD
14	82.1	M	30	95	86.3	4.5		y	61	UNI-H	UNI-N.R.	33	MELAS
14	43.8	M	37	25	25	2.3	y			NOR	BI-N.R.	31	MELAS
9	35.5	F	30	50	43.8	5	y		47	BI-H	BI-N.R.	26	MIDD
5	20.2	M	46	31.3	25	3.25				NE	NE	20	MIDD
3	18.4	F	56	66.3	68.8	3.95				BI-H	UNI-N.R.	47	MIDD

BI-H: bilateral hyporeflexia; BI-N.R.: bilateral no-response; CI: cochlear implantation; cVEMP: cervical vestibular-evoked myogenic potential; F: female; HL: hearing level; M: male; MELAS: mitochondrial encephalomyopathy, lactic acidosis, and stroke-like episodes; MIDD: maternally inherited diabetes and deafness; NE: not examined; NOR: normal; UNI-H: unilateral hyporeflexia; UNI-N.R.: unilateral hyporeflexia; y: yes; y.o.: years old; yr: year.

## Data Availability

The data presented in this study are available on request from the corresponding author.

## Supplementary Materials

The following supporting information can be downloaded at: https://www.mdpi.com/article/10.3390/life12040543/s1, Figure S1: Chronological progression of hearing level from the first visit, Figure S2: Relationship between patients’ age and progression of their hearing loss, Figure S3: Chronological change in hearing level from the first visit in patients who showed rapid decline in hearing.
