# Supplementary material for: Long-Term Progression and Rapid Decline in Hearing Loss in Patients with a Point Mutation at Nucleotide 3243 of the Mitochondrial DNA
Source: Life (Basel). 2022 Apr 6;12(4):543. doi: 10.3390/life12040543 (PMC9033132; doi:10.3390/life12040543)
Supplement: Supplementary file 1 [file life-12-00543-s001.zip › life-1644065-supplementary.pdf]

Supplemental Figure S1,2 and 3

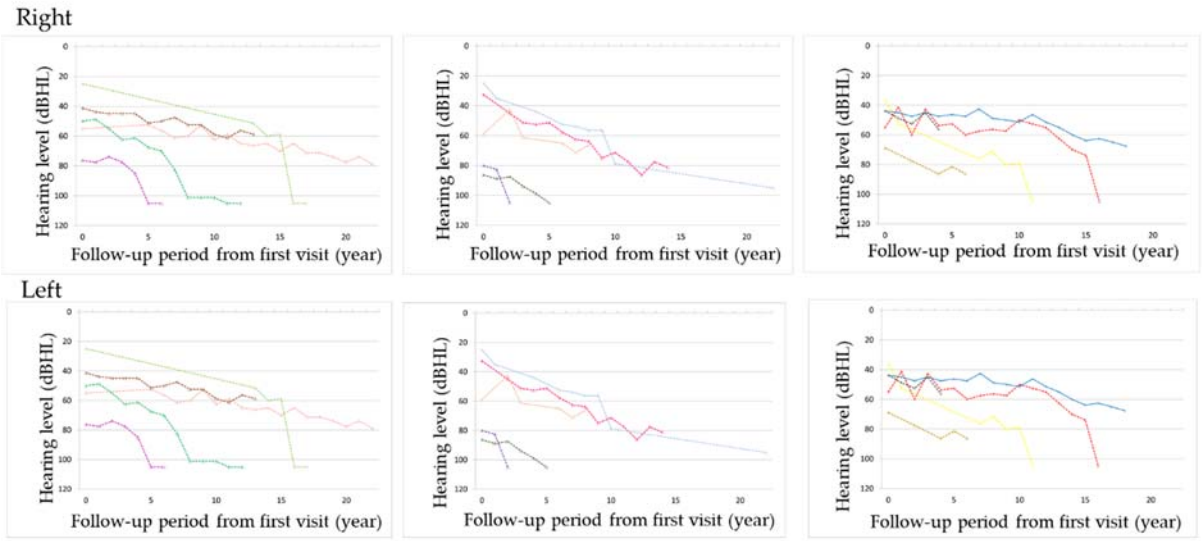

Figure S1: Chronological progression of hearing level from the first visit. To distinguish a line clearly, separated graphs (five patients each) from Figure 1 are shown. The different color indicates the different patient and the same color indicates the same patient.

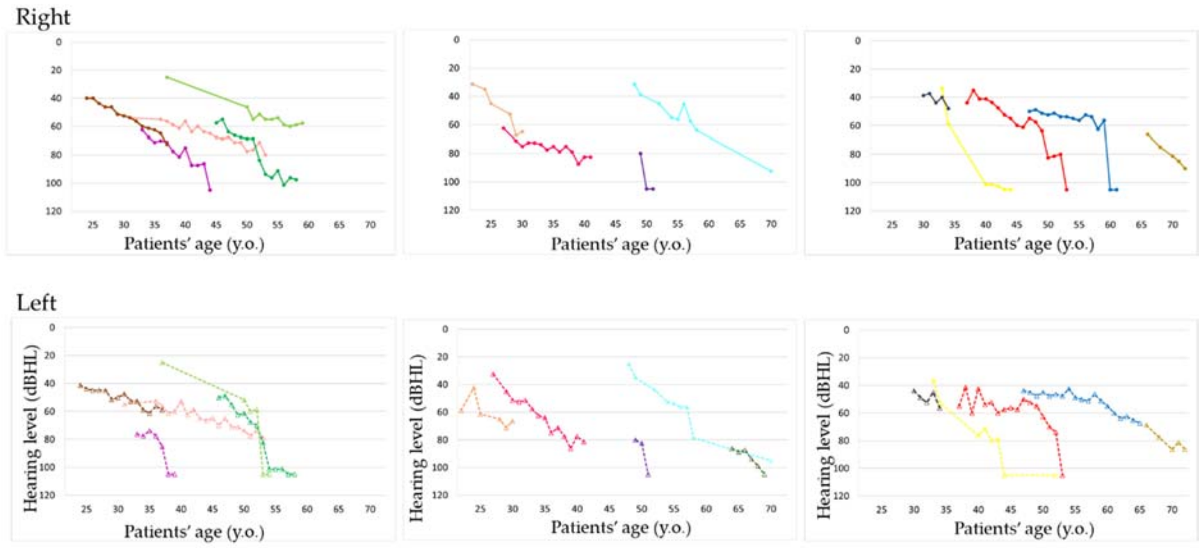

Figure S2: Relationship between patients' age and progression of their hearing loss. Separated graphs (five patients each) from Figure 2 are shown.

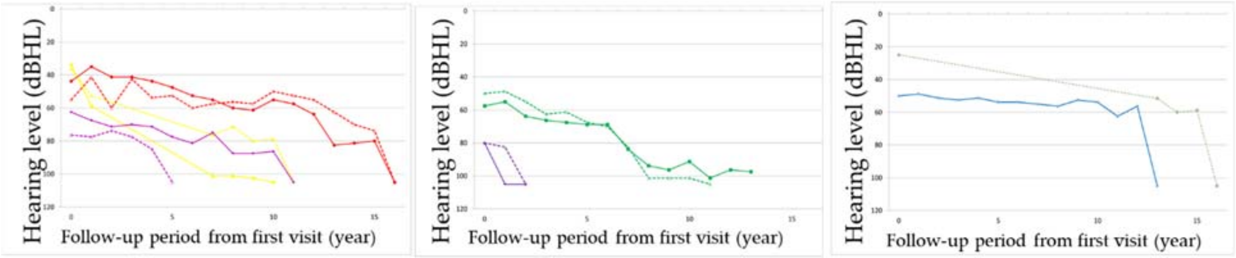

Figure S3: Chronological change of hearing level from the first visit in patients who showed rapid decline of hearing. Separated graphs from Figure 3 are shown. The solid lines represent right ear. The dashed line represents left ear.
